# Impact of smoking on early clinical outcomes in patients undergoing coronary artery bypass grafting surgery

**DOI:** 10.1186/s13019-015-0216-y

**Published:** 2015-02-06

**Authors:** Qiang Ji, Hang Zhao, YunQing Mei, YunQing Shi, RunHua Ma, WenJun Ding

**Affiliations:** 1 Department of Thoracic Cardiovascular Surgery of Tongji Hospital of Tongji University, 389 Xincun Rd, Shanghai, 200065 P.R. China; 2 Master student, Tongji University, 1239 Siping Rd, Shanghai, 200092 P.R. China; 3 Department of Cardiovascular Surgery of Zhongshan Hospital of Fudan University, 180 Fenglin Rd, Shanghai, 200032 P.R. China

**Keywords:** Persistent smoking, Smoking cessation, Early clinical outcomes, Coronary artery bypass grafting surgery

## Abstract

**Background:**

To evaluate the impact of persistent smoking versus smoking cessation over one month prior to surgery on early clinical outcomes in Chinese patients undergoing isolated coronary artery bypass grafting (CABG) surgery in a retrospective study.

**Methods:**

The peri-operative data of consecutive well-documented patients undergoing isolated CABG surgery from January 2007 to December 2013 were investigated and retrospectively analyzed. All included patients were divided into either a non-smoking group or a smoking group according to preoperative smoking records. Furthermore, smokers were divided into either a former smoking subgroup (smokers with smoking cessation over 1 month before surgery) or a current smoking subgroup (persistent smokers).

**Results:**

A total of 3730 consecutive patients (3207 male patients and mean 63.6 ± 9.5 years) undergoing isolated CABG surgery were analyzed. Persistent smokers had significantly higher incidence of postoperative pulmonary complications as compared to non-smokers (7.8% vs. 4.5%, p = 0.0002). No significantly differences in both surgical mortality and major postoperative morbidities between smokers with smoking cessation over 1 month before surgery and non-smokers were found. In multiple logistic regression analysis, the risk of postoperative pulmonary complications in persistent smokers was 2.41 times than that in non-smokers, whereas the risk of postoperative pulmonary complications in smokers with smoking cessation over 1 month before surgery was similar to non-smokers.

**Conclusions:**

Persistent smokers had a higher incidence of pulmonary complications following CABG as compared to non-smokers. Smoking cessation more than 1 month before surgery was expected to reduce early major morbidities following CABG surgery.

## Background

Smoking is a major risk factor for coronary heart disease. Previous study [1] reported smoking cessation has brought about a reduction of incidence of coronary heart disease of 12%. Coronary artery bypass grafting surgery (CABG) is an important means of treatment of coronary heart disease. Warner MA and colleagues [2] conducted a blinded prospective study and showed that smokers with smoking cessation less than 2 months before CABG surgery had a higher incidence of postoperative pulmonary complications as compared to smokers with smoking cessation more than 2 months before CABG surgery. With the progress in surgical techniques and postoperative care, major morbidities following CABG were also on the decline. Recently, it is reported that smoking cessation more than 1 month before CABG procedure evidently reduced pulmonary complications following CABG surgery [3].

So far, China becomes the largest tobacco producer and consumer in the world, with more than 300 million smokers [4]. However, there are few reports about the impact of smoking on clinical outcomes of Chinese patients undergoing isolated CABG surgery. In this retrospective study, we aimed to evaluate the impact of persistent smoking vs. smoking cessation over 1 month before surgery on early postoperative clinical outcomes through reviewing 3730 consecutive Chinese patients undergoing isolated CABG procedure.

## Methods

### Subjects

After approval by the ethics committee of Tongji hospital affiliated to Tongji University, in accordance with the *Declaration of Helsinki*, we reviewed the records of consecutive well-documented patients undergoing either emergency or scheduled isolated CABG procedure with or without cardiopulmonary bypass from January 2007 to December 2013. Any patient with incomplete information from preoperative smoking records was excluded from this study. According to smoking records before CABG surgery, all included patients were divided into either a smoking group (smokers) or a non-smoking group (non-smokers). Furthermore, the smoking group (smokers) was divided into either a former smoking subgroup (smokers with smoking cessation over 1 month before CABG surgery) or a current smoking subgroup (persistent smokers). Data collection was performed by trained staff (two people), and they did not know the purpose of this study.

The details of the coronary artery lesions (SYNTAX score [5]), in association with the preoperative characteristics, was carefully evaluated for assignment to CABG with (on-pump CABG) or without cardiopulmonary bypass (off-pump CABG). In every patient complete anatomic revascularization of all diseased vessels with a luminal diameter greater than or equal to 1 mm was considered necessary. If the operating surgeon judged a complete revascularization feasible on the beating heart, off-pump CABG was scheduled. In patients in whom the location or the quality of the target vessels and the preoperative characteristics (such as left ventricular end diastolic dimension, left ventricular ejection fraction, etc.) was considered to make off-pump revascularization technically too challenging, on-pump CABG was scheduled. All the procedures (either off-pump or on-pump CABG) were performed by the same surgical team and there were no differences in the rate of adoption of off-pump CABG between the different surgeons. In our center, off-pump CABG had been performed routinely for >5 years before the launch of the trial, and all of the operations were performed by 3 surgeons highly experienced in both off-pump and on-pump surgery (each of the 3 participating surgeons performed at least 50% of their CABG procedures as off-pump CABG).

### Definition of surgical mortality and major postoperative morbidities

Surgical mortality was defined as death that occurred during the same hospitalization or within 30 days of CABG surgery [6]. Major postoperative morbidities included postoperative myocardial infarction, low cardiac output syndrome (LCOS), new onset atrial fibrillation, cardiac arrest or ventricular fibrillation, pulmonary complications, renal failure, stroke, gastro-intestinal complications, incision infection, and multiple system organ failure (MSOF). Postoperative myocardial infarction was defined by either the appearance of new Q waves in 2 or more contiguous leads on the electrocardiogram, or an increase in the creatine kinase MB isoenzyme fraction of more than 50U, in concert with an excess of 7% of the total creatinine kinase level [7]. LCOS was considered with those who met the following criteria before discharge from first hospitalization in the intensive care unit immediately after CABG surgery: 1. Need for inotropic support with vasoactive drugs or mechanical circulatory support with intra-aortic balloon pump to maintain systolic blood pressure greater than 90 mmHg after correction of all electrolytes and blood gas abnormalities while adjusting preload volume to its optimal values; 2. Signs of impairment of body perfusion after correction of all electrolytes and blood gas abnormalities while adjusting preload volume to its optimal values [8]. New onset atrial fibrillation was considered as postoperative intermittent or persistent new onset atrial fibrillation before discharge. Cardiac arrest or ventricular fibrillation was defined as postoperative unexplained heart arrest or ventricular fibrillation before discharge, resulting in loss of cardiac ejection function, and requiring cardiopulmonary resuscitation. Pulmonary complications included postoperative pneumonia which was defined as a positive result in a sputum culture requiring anti-infective treatment, or chest X-ray diagnosis of pneumonia following CABG surgery [9] and postoperative respiratory failure which was defined as the duration of mechanical ventilation more than 48 h after surgery or unplanned re-intubation within 30 days of surgery [10] (although some previous reports defined postoperative respiratory failure as requiring mechanical ventilation > 72 h or re-intubation following surgery, this study defined postoperative respiratory failure as requiring mechanical ventilation > 48 h or re-intubation following surgery in view of the majority of included patients undergoing off-pump CABG). Renal dysfunction was defined as serum creatinine greater than 2.5 mg/dl for more than 7 days or requiring dialysis [11]. Stroke was defined as a new focal or global dysfunction of cerebral function lasting over 24 h. Gastro-intestinal complications included gastrointestinal bleeding, intestinal obstruction and other serious gastrointestinal dysfunction. Incision infection meant the chest or leg wound infection requiring antibiotic therapy or operation [12]. MSOF (a severe dysfunction of at least two organ systems lasting for more than 24 h, as stated by the Consensus Conference of the American College of Chest Physicians and the Society of Critical Care Medicine [13]) was recorded. In addition, duration of mechanical ventilation and length of intensive care unit (ICU) stay were also recorded.

### Statistics analysis

Statistical analysis was performed using the SPSS 17.0 statistical software package. All p values <0.05 were considered to be statistically significant. Normal distribution of measurement data was shown by mean ± standard deviation, and was compared between the groups by *t*-test. *Fisher’s* exact test was employed to compare enumeration data. In order to further confirm the reliability of results, mortality and major postoperative morbidities were further analyzed by multiple logistic regression analysis. The covariate of regression analysis included age, gender, body mass index, previous disease history (included diabetes, hyperlipaemia, hypertension, chronic obstructive pulmonary disease, myocardial infarction, and peripheral vascular disease), left ventricular ejection fraction, application of the internal mammary artery, use of cardiopulmonary bypass, and number of grafts.

## Results

### Study population

From January 2007 to December 2013, after obtaining approval by the ethic committee, a total of 3856 consecutive patients undergoing isolated CABG were entered into this study. One hundred and twenty-six patients were excluded due to incomplete information from preoperative smoking records, and finally 3730 patients (3207 male patients and mean 63.6 ± 9.5 years) were retrospectively analyzed. According to preoperative smoking records, 2186 patients (accounting for 58.6%) who had more than 1 cigarette every day with continuing at least 1 year were included into a smoking group, and the other 1544 patients without a history of smoking into a non-smoking group. And then, the smoking group was divided either into a former smoking subgroup (smokers with smoking cessation over 1 month before CABG surgery, n = 614) or a current smoking group (persistent smokers, n = 1572).

Baseline data of all included patients are shown in Table 1. Comparing with the non-smoking group, the smoking group was more likely to present with male (98.6% vs. 68.1%, p < 0.0001), history of myocardial infarction (53.0% vs. 46.7%, p < 0.0001), chronic obstructive pulmonary disease (COPD) (14.0% vs. 6.1%, p < 0.0001), and high body mass index (BMI) (25.1 ± 3.1 kg/m^2^ vs. 24.8 ± 3.2 kg/m^2^, p = 0.0041), but was less likely to hypertension (50.5% vs. 58.1%, p < 0.0001) and hyperlipidemia (31.3% vs. 38.7%, p < 0.0001). Patients in the smoking group was younger (62.2 ± 9.4 years vs. 65.7 ± 9.7 years, p < 0.0001) and had lower left ventricular ejection fraction (LVEF) (57.9 ± 9.9% vs. 59.7 ± 9.0%, p < 0.0001) as compared to the non-smoking group. And comparing with the non-smoking group, the former smoking subgroup was more likely to present with male (98.0% vs. 68.1%, p < 0.0001), history of myocardial infarction (51.9% vs. 46.7%, p = 0.0029), COPD (15.8% vs. 6.1%, p < 0.0001), and high BMI (25.2 ± 3.0 kg/m^2^ vs. 24.8 ± 3.2 kg/m^2^, p = 0.0017), but was younger (60.7 ± 8.8 years vs. 65.7 ± 9.7 years, p < 0.0001) and had lower LVEF (57.5 ± 9.7% vs. 59.7 ± 9.0%, p < 0.0001). In addition, the current smoking subgroup was more likely to present with male (98.9% vs. 68.1%, p < 0.0001), history of myocardial infarction (53.4% vs. 46.7%, p = 0.0002) and COPD (13.3% vs. 6.1%, p < 0.0001), but was less likely to hypertension (48.3% vs. 58.1%, p < 0.0001) and hyperlipidemia (30.0% vs. 38.7%, p < 0.0001) as compared to the non-smoking group. Comparing with the non-smoking group, the current smoking subgroup was less likely to undergoing CABG with cardiopulmonary bypass (17.6% vs. 27.1%, p < 0.0001) and had lower LVEF (58.1 ± 9.8% vs. 59.7 ± 9.0%, p < 0.0001).

**Table 1:** **Baseline data of all included patients**

Variables	Non-smoking group	Smoking group		
		Total	Former smoking subgroup	Current smoking subgroup
	(n = 1544)	(n = 2186)	(n = 614)	(n = 1572)
Age (years)	65.7 ± 9.7	62.2 ± 9.4^*^	60.7 ± 8.8^*^	65.3 ± 9.2
Male	1051 (68.1%)	2156 (98.6%)^*^	602 (98.0%)^*^	1554 (98.9%)^*^
BMI (kg/m^2^)	24.8 ± 3.2	25.1 ± 3.1^*^	25.2 ± 3.0^*^	24.9 ± 3.1
Familial history of CAD	1425(92.3%)	1994(91.2%)	556(90.6%)	1438(91.5%)
Diabetes mellitus	559(36.2%)	783(35.8%)	230(37.5%)	553(35.2%)
Hypertension	897(58.1%)	1103 (50.5%)^*^	344(56.0%)	759 (48.3%)^*^
Hyperlipemia	597(38.7%)	684 (31.3%)^*^	212(34.5%)	472 (30.0%)^*^
COPD	94 (6.1%)	306 (14.0%)^*^	97 (15.8%)^*^	209 (13.3%)^*^
History of MI	721(46.7%)	1158 (53.0%)^*^	319 (51.9%)^*^	839(53.4%)^*^
Prior CVA	240(15.5%)	348(15.9%)	108(17.6%)	240(15.3%)
Coronary artery lesion				
Double-vessel	134(8.7%)	198 (9.0%)	54 (8.8%)	144(9.1%)
Triple-vessel	1408(91.2%)	1985(90.8%)	558(90.9%)	1427(90.8%)
Left main	378(24.5%)	529 (24.2%)	147 (23.9%)	382 (24.3%)
SYNTAX score				
Low: ≤ 22	278 (18.0%)	306 (14.0%)	101 (16.4%)	200 (13.1%)
Intermediate: 23-32	793 (51.4%)	1194 (54.6%)	324 (52.8%)	870 (55.3%)
High: ≥33	473 (30.6%)	686 (31.4%)	189 (30.8%)	497 (31.6%)
LVEF (%)	59.7 ± 9.0	57.9 ± 9.9^*^	57.5 ± 9.7^*^	58.1 ± 9.8^*^
>50%	1275 (82.6%)	1631 (74.6%)^*^	463 (75.4%)^*^	1168 (74.3%)^*^
35-50%	178 (11.5%)	407(18.6%)^*^	99 (16.1%)^*^	308 (19.6%)^*^
<35%	91 (5.9%)	148 (6.8%)^*^	52(8.5%)^*^	96 (6.1%)
Emergence operation	123 (8.0%)	179 (8.2%)	49 (7.9%)	130 (8.3%)
On-pump CABG	418(27.1%)	439 (20.1%)^*^	163(26.5%)	276 (17.6%)^*^
CPB (min)	78.2 ± 31.4	79.6 ± 27.8	75.5 ± 24.8	80.1 ± 28.4
ACC (min)	57.2 ± 20.6	58.5 ± 19.5	57.4 ± 18.6	59.4 ± 20.6
Use of IMA	1518 (98.3%)	2144 (98.1%)	603 (98.2%)	1541 (98.0%)
Number of grafts	3.3 ± 0.5	3.3 ± 0.4	3.3 ± 0.6	3.3 ± 0.4

*Fisher’s* exact test was employed to compare enumeration data, and *t*-test to compare measurement data.

*p < 0.05 (as compared to Non-smoking group).

BMI, body mass index; CAD, coronary artery disease; COPD, chronic obstructive pulmonary disease; MI, myocardial infarction; CVA, cerebro-vascular accident; LVEF, Left ventricular ejection fraction; CABG, coronary artery bypass grafting; On-pump CABG, CABG with cardiopulmonary bypass; CPB, cardiopulmonary bypass; ACC, aortic cross clamp; IMA, internal mammary artery.

### Early outcomes following CABG

The comparison of surgical mortality and major postoperative morbidities between the smoking group and non-smoking group is shown in Table 2. A total of 92 patients were died, with a surgical mortality of 2.5%. The smoking group had a low surgical mortality as compared to the non-smoking group, but no statistical difference was found (2.2% vs. 2.8%, p = 0.3348). The smoking group had significantly higher incidence of postoperative pulmonary complications (7.1% vs. 4.5%, p = 0.0010), and significantly longer duration of mechanical ventilation (9.8 ± 2.6 h vs. 9.4 ± 2.4 h, p < 0.0001) as compared to the non-smoking group. There were no statistically significant differences between the smoking group and the non-smoking group in postoperative myocardial infarction, low cardiac output syndrome, new onset atrial fibrillation, cardiac arrest or ventricular fibrillation, acute kidney injury, stroke, gastro-intestinal complications, incision infection, and multiple system organ failure.

**Table 2:** **Early clinical outcomes after CABG surgery**

	Non-smoking group (n = 1544)	Smoking group		
		Total	Former smoking subgroup	Current smoking subgroup
		(n = 2186)	(n = 614)	(n = 1572)
Surgical mortality	43(2.8%)	49(2.2%)	18(2.9%)	31(2.0%)
MI	16(1.7%)	24(1.1%)	8(1.3%)	16(1.0%)
LCOS	282(18.3%)	433(19.8%)	124(20.2%)	309(19.7%)
New onset AF	177(11.5%)	227(10.4%)	78(12.7%)	95(9.5%)
Ventricular fibrillation	29(1.9%)	46(2.1%)	15(2.4%)	31(2.0%)
Pulmonary complications	70 (4.5%)	156 (7.1%)^*^	34 (5.5%)	122 (7.8%)^*^
Mean ventilation time (h)	9.4 ± 2.4	9.8 ± 2.6^*^	9.5 ± 2.3	9.9 ± 2.7^*^
Renal failure	38(2.5%)	53(2.4%)	14(2.3%)	39(2.5%)
Stroke	31(2.0%)	20(0.9%)	5(0.8%)	15(1.0%)
GI complications	82(5.3 %)	120(5.5%)	19(3.1%)	101(6.4%)
Incision infection	21(1.4%)	33(1.5%)	9(1.5%)	24(1.5%)
MSOF	25(1.6%)	29(1.3%)	6(1.0%)	23(1.5%)
Mean length of ICU stay (h)	33.5 ± 6.9	34.2 ± 6.8	33.5 ± 6.1	34.5 ± 7.0

Mean was compared between the groups by *t-*test, and enumeration data by *Fisher’s* exact test.

*p < 0.05 (as compared to Non-smoking group).

MI, myocardial infarction; LCOS, low cardiac output syndrome; AF, atrial fibrillation; GI complications, gastro-intestinal complications; MSOF, multiple system organ failure; ICU, intensive care unit.

The comparison of surgical mortality and major postoperative morbidities between the current smoking subgroup and non-smoking group is shown in Table 2. There was no significant difference in surgical mortality between the 2 groups (2.0% vs. 2.8%, p = 0.1579). Comparing with the non-smoking group, the current smoking subgroup had significantly higher incidence of postoperative pulmonary complications (7.8% vs. 4.5%, p = 0.0002) and significantly longer duration of mechanical ventilation (p < 0.001). There were no statistically significant differences between the two groups in postoperative myocardial infarction, low cardiac output syndrome, new onset atrial fibrillation, cardiac arrest or ventricular fibrillation, acute kidney injury, stroke, gastro-intestinal complications, incision infection, and multiple system organ failure.

The comparison of early outcomes following CABG between the former smoking subgroup and the non-smoking group is shown in Table 2. There were no statistically significant differences between the 2 groups in surgical mortality, major postoperative morbidities, and length of mechanical ventilation and ICU stay.

### Logistic regression analysis

The impact of smoking on surgical mortality and major postoperative morbidities after adjustment for potential confounders (age, gender, BMI, diabetes, hyperlipaemia, hypertension, COPD, recent myocardial infarction, peripheral vascular disease, LVEF, application of internal mammary artery, use of cardiopulmonary bypass, number of grafts) is shown in Table 3. In multivariate logistic regression analysis, smoking had an independent influence on the development of postoperative pulmonary complications (OR = 1.92, 95% CI 1.08-3.64). And, multivariate logistic regression analysis also showed that the risk of postoperative pulmonary complications in persistent smokers was 2.41 times than that in non-smokers, whereas the risk of postoperative pulmonary complications in smokers with smoking cessation over 1 month before CABG was similar to non-smokers.

**Table 3:** **Logistic regression analysis**

Events	Non-smoking group	Smoking group		
		Total	Former smoking subgroup	Current smoking subgroup
		OR (95% CI)	OR (95% CI)	OR (95% CI)
Surgical mortality	1.0	1.10 (0.42-2.73)	0.85 (0.31-2.42)	1.41 (0.42-3.91)
MI	1.0	1.23 (0.48-3.12)	1.12 (0.43-2.89)	1.45 (0.63-3.49)
LCOS	1.0	1.13 (0.82-1.51)	1.22 (0.82-1.84)	1.05 (0.85-1.38)
New onset AF	1.0	1.07 (0.74-1.61)	0.95 (0.62-1.62)	1.41 (0.77-2.21)
Ventricular fibrillation	1.0	1.43 (0.65-3.14)	1.93 (0.78-4.82)	1.31 (0.58-2.95)
Pulmonary complications	1.0	1.92 (1.08-3.64)^*^	1.32 (0.61-3.16)	2.41 (1.21-4.64)^*^
Renal failure	1.0	1.20 (0.54-2.63)	1.05 (0.41-2.97)	1.38 (0.52-3.10)
Stroke	1.0	0.92 (0.41-2.14)	0.41 (0.11-1.83)	0.29 (0.08-1.31)
GI complications	1.0	1.55 (0.45-4.75)	0.51 (0.11-3.76)	1.79 (0.55-5.48)
Incision infection	1.0	1.31 (0.44-4.78)	2.11 (0.55-8.93)	1.17 (0.29-5.14)
MSOF	1.0	1.14 (0.44-3.10)	0.62 (0.21-2.99)	1.51 (0.48-4.03)

*p < 0.05 (as compared to Non-smoking group).

## Discussion

Although the public gradually raised awareness of the dangers of smoking and Chinese government attached great importance to the tobacco control, the prevalence rate of smoking in China remains unacceptably high in the current era [4]. The prevalence rate of smoking in patients with coronary artery disease undergoing isolated CABG surgery in this study touched 58.6%, with far above 30% in the Western countries reports [14-16], suggesting that the task of tobacco control was difficult, and moreover, time was pressing. There were rare reports about the impact of smoking on clinical outcomes in Chinese patients undergoing isolated CABG. Based on these, this study aimed to evaluate the impact of persistent smoking vs. smoking cessation over 1 month before surgery on early postoperative clinical outcomes by reviewing 3730 Chinese patients with or without a history of smoking undergoing isolated CABG surgery.

The major finding of this study was that comparing with non-smokers, persistent smokers were more likely to suffer from postoperative pulmonary complications, whereas smokers with smoking cessation over 1 month before CABG surgery did not increase the incidence of postoperative pulmonary complications. In this study, univariate analyses showed that persistent smokers had a higher incidence of postoperative pulmonary complications (7.8% vs. 4.5%, p = 0.0002) as compared to non-smokers, whereas smokers with smoking cessation over 1 month before CABG surgery and non-smokers shared a similar incidence of postoperative pulmonary complications (5.5% vs. 4.5%, p = 0.3183). Furthermore, multiple logistic regression analysis showed that the risk of postoperative pulmonary complications in persistent smokers was 2.41 times than that in non-smokers, whereas the risk of postoperative pulmonary complications in those smokers with smoking cessation over 1 month before CABG was similar to non-smokers. Reason of this difference may be that smoking caused obstructive ventilation function disturbance and aggravated respiratory disease, contributing to postoperative pulmonary complications, whereas smoking cessation over 1 month may gradually improve ciliary function and macrophage phagocytic function, and steadily decreased the sputum, resulting in the reduction of postoperative pulmonary complications in a few weeks [17,18]. This result was consistent with previous studies [2,3,14,15,17,18]. In addition, smokers with smoking cessation over 1 month before CABG surgery and non-smokers shared a similar incidence of major postoperative morbidities, suggesting that smoking cessation more than 1 month before CABG surgery was expected to reduce early major morbidities after CABG surgery and to accelerate recovery.

This study showed smokers were less likely to hypertension and hyperlipidemia as compared to non-smokers. Reason of this difference may be the age, regarding that smokers suffered with coronary atherosclerosis received treatment in their youth, and thus were less likely to suffering from senile diseases, such as hypertension, hyperlipidemia [19]. In this study, smokers were more likely to history of myocardial infarction and had lower left ventricular ejection fraction as compared to non-smokers. Higher incidence of myocardial infarction, in association with reduction of cardiac function, may lead to more serious condition, hence may unfavourable for postoperative recovery. This study also showed that there was no significant difference in surgical mortality between smokers and non-smokers, which was consistent with Al-Sarraf N’s study [20]. Smokers were younger and had lower incidence of hypertension and hyperlipidemia, but had higher incidence of chronic disease obstructive pulmonary disease and history of myocardial infarction with poor cardiopulmonary function, and then had the similar surgical mortality to non-smokers [3,14].

So far, few studies have assessed the relationship between persistent smoking vs. smoking cessation before surgery and early clinical outcomes of Chinese patients undergoing isolated CABG surgery. This study showed that persistent smoking increased postoperative pulmonary complications, which not only prolonged rehabilitation, but also may cause other morbidities, consequently increasing the financial burden. Smoking cessation over 1 month before CABG surgery was expected to reduce early major morbidities after CABG surgery.

There are some limitations of this study. This study was only a retrospective observational trial, which may influence the generalizability. Secondly, relatively low postoperative morbidities and mortality in this study may cause false negative result, which remained to be resolved by expanding the sample size. Finally, this study focused on the early clinical outcomes, and further follow-up study involving larger sample size were needed to determine the effect of smoking on long-term clinical outcomes after CABG surgery.

## Conclusions

Persistent smokers had a higher incidence of pulmonary complications following CABG as compared to non-smokers. Smoking cessation more than 1 month before CABG surgery was expected to reduce early major morbidities after CABG surgery.

## Abbreviations

CABG: Coronary artery bypass grafting
LCOS: Low cardiac output syndrome
MSOF: Multiple system organ failure
ICU: Intensive care unit
COPD: Chronic obstructive pulmonary disease
BMI: Body mass index
LVEF: Left ventricular ejection fraction
OR: Odds ratio
